# Influence of Sulfates on Formation of Ettringite during Early C_3_A Hydration

**DOI:** 10.3390/ma15196934

**Published:** 2022-10-06

**Authors:** Yong Yang, Qianqian Zhang, Xin Shu, Xiumei Wang, Qianping Ran

**Affiliations:** 1 School of Materials Science and Engineering, Southeast University, Nanjing 211189, China; 2 State Key Laboratory of High Performance Civil Engineering Materials, Jiangsu Sobute New Materials Co., Ltd., Nanjing 210008, China

**Keywords:** specific surface area, hydration, ettringite, hemihydrate, C_3_A

## Abstract

The hydration of C_3_A-gypsum systems was studied in the presence of various types of sulfates such as gypsum, hemihydrate and Na_2_SO_4_ in the first hour. The BET method combined with a DSC analysis enabled us to quantitatively characterize the amount of precipitated ettringite and its specific surface area along the hydration. It was found that sulfates not only affected the formation rate of ettringite, but also had a significant impact on the morphology of ettringite. For all the C_3_A-gypsum systems, a large part of the ettringite precipitated within the first 20 min and the specific surface area of the hydrated sample strongly increased within the first 5 min, whereas the specific surface area of ettringite gradually decreased along the C_3_A hydration reaction. Incorporating a small amount of Na_2_SO_4_ in the C_3_A-gypsum system could greatly promote the formation rate of ettringite in the first 20 min, and significantly decrease the specific surface area of ettringite. As hemihydrate was added to the C_3_A-gypsum system, two processes of ettringite precipitation and gypsum precipitation occurred. The nucleation and growth process of ettringite and gypsum resulted in the complex changes in the specific surface area of the hydrated sample, which first increased at the very beginning, then decreased and, finally, increased.

## 1. Introduction

Tricalcium aluminate (C_3_A) is one of the main components of Portland cement. Although the content of this phase is just only 2–10% of the mass of cement, C_3_A has a very significant impact on the workability of cement paste. C_3_A strongly reacts with water and could lead to the “flash-setting” of cement. To avoid this problem, gypsum (CaSO_4_⋅2H_2_O) is usually added as an additive during cement production to regulate the hydration of C_3_A, which allows for more working time with the fresh cement paste as well as with the concrete [1,2,3,4,5].

At present, a large number of studies have focused on the reaction process of C_3_A-gypsum. Ettringite is considered to be the major hydrate product precipitated during the first hour in the presence of gypsum [6,7,8,9,10]. Meredith et al. [11] and Kong et al. [12] proposed that the formation of ettringite in the early stage resulted in a large increase in the specific surface area of solid particles in paste. Pourchet et al. [13] and Zhang et al. [14] found that the morphology of precipitated ettringite changed over time. These results indicate that the formation of ettringite could change particle packing, change the inter-particle distance, and then, change the particle network structure [15], which in turn results in a change in the rheological properties of paste. Therefore, it is crucial to understand the changes in rheological properties of paste over time to discuss the reaction process of C_3_A-gypsum systems.

However, not only gypsum, but also hemihydrate and soluble sulfates (e.g., Na_2_SO_4_ and K_2_SO_4_) can be found in cement due to some factors such as mineral raw materials and the production processes of cement. It can be concluded, based on much research, that the hydration rate of C_3_A was closely related to the type of sulfate sources used, which determines the solubility and rate of dissolution of sulfates used [16,17,18,19,20,21]. Bensted et al. [16] found that the high-temperature grinding of cement resulted in the partial dehydration of gypsum to form hemihydrate, and then induced an increase in the precipitation rate of ettringite in the first 2 h because hemihydrate had a higher solubility and dissolution rate than gypsum. Pourchet et al. [22] suggested that the introduction of hemihydrate as a replacement led to an increase in the formation rate of ettringite during at least the first five hours. In addition, soluble sulfates significantly affect the formation of ettringite. Feng et al. [23] found that ettringite precipitated in the C_3_A-Na_2_SO_4_ system was less than that in the C_3_A-gypsum system in the first hour, and the morphology of ettringite slightly changed. As mentioned above, C_3_A hydration is known to significantly affect rheological properties of cement paste, and thus, of concrete. It is important to study the changes caused by different sulfates during the early C_3_A hydration, which is of great significance for better understanding the time-dependent rheological properties of pastes prepared from cement with different characteristics.

The main aim of this work is to understand the effect of sulfates on the hydration kinetics of C_3_A (formation of ettringite) and the morphology of hydration products. Focusing on the C_3_A-gypsum systems, the effects of sulfate types (gypsum, hemihydrate, Na_2_SO_4_) and proportions on the C_3_A hydration in the first hour were systematically studied. Considering that the superplasticizer affected the formation rate and morphology of ettringite [13], all studies were carried out in the presence of a superplasticizer, which was closer to the actual application environment of cement. In the study, the BET method using nitrogen combined with a DSC analysis was adopted to simultaneously characterize the amount of precipitated ettringite and its specific surface area at a certain time during the hydration process of C_3_A-gypsum.

## 2. Materials and Methods

### 2.1. Minerals

In this study, high-purity C_3_A was prepared by sintering compacted powders of calcium hydroxide as the calcium raw material and alumina as the aluminum raw material. The sintering temperature was 1350 °C and the holding time was 4 h. The pellets of C_3_A needed to be sintered twice with an intermediate grinding to ensure homogeneity and a complete reaction. Finally, the powders were ground and sieved, and particles <45 μm were retained. Based on the designed research plan, gypsum (G), hemihydrate (H) or Na_2_SO_4_ (S) was added to the powders to obtain the final composition. A commercial comb-shaped polycarboxylate superplasticizer (PC) compounded with a defoaming agent provided by Jiangsu Sobute New Materials Co., Ltd. was used.

In this study, five groups of simulated solutions with different SO_4_^2−^ concentrations were prepared by Na_2_SO_4_ and NaNO_3_, as shown in Table 1. The ionic strength of these simulated solutions was similar, which avoided the influence of ionic strength on the hydration of the C_3_A-gypsum system. Certain amounts of Na_2_SO_4_ and NaNO_3_ were dissolved in deionized water. These solutions were continuously stirred for 6 h at 20 °C and then filtered through 0.3μm Millipore filters.

### 2.2. Methods

#### 2.2.1. Preparation of the Suspensions

All the experiments were carried out with a liquid/solid ratio equal to 1 and PC dosage of 0.1%. The experiments in this study could be divided into two groups, and the mixture proportions and codes are shown in Table 2 and Table 3. The experiments about the effect of the ratio of C_3_A/G in the C_3_A-gypsum system and the SO_4_^2−^ concentration in the simulated solutions on the hydration of the C_3_A-gypsum system at 20 min were systematically carried out (as shown in Table 2). The mixture proportions listed in Table 3 involved the effect of the SO_4_^2−^ concentration in simulated solutions (0, 0.015, 0.06 mol/L) and replacing gypsum with quality hemihydrate gypsum (10%, 20%) or Na_2_SO_4_ in a solid (1.7%, 3.4%) on C_3_A-gypsum hydration in the first hour. When considering the effect of hemihydrate and Na_2_SO_4_, deionized water was used as the mixing water.

The paste was prepared using a constant temperature magnetic stirrer according to the following procedure, which could not only stir the pastes, but also control the temperature of the experimental environment at 20 °C. A certain amount of PC together with deionized water or simulated solution was first put into the mixer and mixed for 1 min at a speed of 150 rpm; then, the powder previously prepared according to the mixture proportions listed in Table 2 and Table 3 was added and mixed at a speed of 300 rpm for 2 min. Finally, the paste was mixed at a speed of 150 rpm. Part of the paste was taken out at this time to stop hydration, and the remaining part was continuously mixed until the end of the experiment (20 min or 60 min).

#### 2.2.2. Stopping C_3_A Hydration

C_3_A hydration was stopped for microscopic performance analyses such as DSC analysis, BET nitrogen analysis and analysis of the morphology of hydration products at different times (5 min, 20 min and 60 min). C_3_A hydration was efficiently stopped by a solvent exchange, which could remove capillary water. At each time, about 6 g of the hydrating cement paste was taken out and poured into a cup with 150 g of cold isopropanol at 5 °C (AR ≥ 99.7%). After 1 min of mixing, the suspension was filtered with a polyamide membrane filter (pore size of 0.45 μm) to obtain the solid part, which was then dried under a nitrogen flow in a desiccator at 20 °C under atmospheric pressure until a constant weight [24,25,26]. The dried powder was, then, gently homogenized in an agate mortar before microscopic performance measurements.

#### 2.2.3. DSC Analysis

In the hydrated C_3_A-gypsum systems, there are obvious differences in peaks between ettringite and gypsum. The amount of ettringite in the hydrated sample could, thus, be quantified via differential scanning calorimetry (DSC 214, NETZSCH, Selb, Germany). During the test, the hydrated samples were heated from 25 °C to 200 °C at a rate of 10 °C/min. Even in the samples considering the effect of hemihydrate, no peak of hemihydrate was found in the 5 min hydrated samples due to the extremely fast solution rate of hemihydrate. The only peaks that could be detected are ettringite and gypsum. The amount of ettringite could be determined by the peak area. The temperature interval [25–125 °C] was selected for the ettringite [13]. The amount of ettringite in a hydrated sample was obtained by the ratio of the ettringite peak area per gram of the sample to the peak area for 1 g of pure synthetic ettringite (ΔHE = 960 J/g) measured under the same conditions.

#### 2.2.4. BET Method Using N_2_

The BET method using nitrogen (N_2_) was adopted to measure the specific surface area (SSA) of the previously “stopped” samples. A nitrogen adsorption test was carried out via a TristarII3020 apparatus (Micromeritics, Norcross, GA, USA) from Micromeritics. Before the nitrogen adsorption test, the previously “stopped” sample was degassed in an external degassing station (VacPrep 061 from Micromeritics). Flatt [26] reported that degassing conditions (temperature, pressure and time) were essential for the SSA measurement by nitrogen adsorption, and could lead to the decomposition of ettringite. In this study, all the samples were degassed under a N_2_ flow for 16 h at 40 °C, which was considered to have little effect on ettringite.

#### 2.2.5. Scanning Electron Microscopy (SEM)

An FEI Quanta 250 SEM microscope (FEI, Hillsboro, OH, USA) was used to study the morphology of ettringite. For microscopy, a very small amount of the previously “stopped” sample was evenly dispersed with a constant amount of cold isopropanol (5 °C) in a beaker. A drop of the cement–isopropanol suspension was dropped on a silicon wafer and dried under the nitrogen flow in a desiccator at 20 °C and under atmospheric pressure. Once dried, the sample was prepared for SEM observation.

## 3. Results and Discussion

### 3.1. Effect of Sulfates on the C_3_A Hydration Kinetics

In the presence of sufficient gypsum, ettringite is considered to be the only product during the early stage of C_3_A hydration (the first hour) [22], and the reaction could be described as follows:

$$Ca_{3}Al_{2}O_{6}+3CaSO_{4}+32H_{2}O\to Ca_{6}Al_{2}{\left(SO_{4}\right)}_{3}{\left(OH\right)}_{12}\cdot 26H_{2}O$$

**Figure 1:**
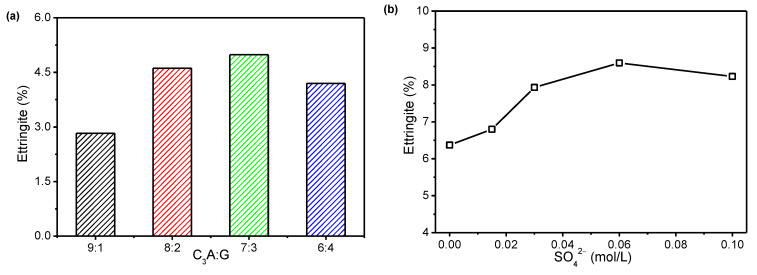
Amount of precipitated ettringite at 20 min: (**a**) effect of C_3_A/G; (**b**) effect of SO_4_^2−^ concentration in simulated solution.

Firstly, different ratios of C_3_A/G and a series of simulated solutions with different concentrations of SO_4_^2−^ were considered for a better understanding of the C_3_A hydration reaction. As a large part of the ettringite was formed in the first 20 min [13], only the C_3_A hydration reaction at 20 min was tested. Based on the DSC data, the amount of ettringite precipitated could be calculated, and the results are shown in Figure 1. The amount of ettringite precipitated at 20 min increased first and then decreased when the proportion of gypsum in the C_3_A-gypsum system increased. When the C_3_A/G was 70/30, the amount of ettringite was the highest. It was considered that a high proportion of gypsum led to a large reduction in the C_3_A content in the C_3_A-gypsum system and then reduced the formation of ettringite. For the hydration of C_3_A in simulated solutions with different SO_4_^2−^ concentrations, the amount of ettringite at 20 min first increased and then decreased with the increasing SO_4_^2−^ concentration. When the SO_4_^2−^ concentration was 0.06 mol/L, the most ettringite was found.

**Figure 2:**
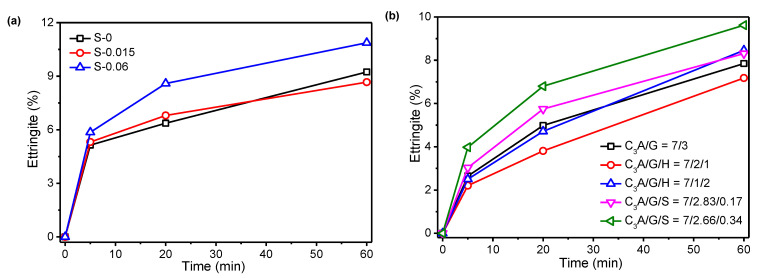
Amount of precipitated ettringite during the first hour: (**a**) effect of SO_4_^2−^ concentration in simulated solution; (**b**) effect of hemihydrate and Na_2_SO_4_ in solid.

Figure 2 shows the amount of precipitated ettringite during the first hour. Regardless of the effect of sulfate concentrations in the simulated solution, or hemihydrate and Na_2_SO_4_ in a solid, a large amount of ettringite was rapidly formed within the first 20 min of C_3_A hydration, whereas the formation rate of ettringite significantly slowed down after 20 min, which was in good agreement with other published results. In fact, the kinetics of the formation of ettringite are still under investigation [5,17,27,28]. However, the amount of ettringite in the hydrated sample calculated from the DSC data was consistent with the finding that this was a two-step reaction: quickly, C_3_A hydration led to a large amount of precipitated ettringite in the first minutes, and after that, the C_3_A hydration was delayed due to the newly generated surface of hydrates.

Figure 2a shows that the SO_4_^2−^ concentration of 0.015 mol/L in a simulated solution with an equivalent ionic strength only slightly modified the reaction rate of the C_3_A hydration, but when the concentration increased to 0.06 mol/L, the amount of precipitated ettringite was significant increased. The presence of SO_4_^2−^ in the solution seemed to promote the formation of ettringite in the first 20 min, and reduce the rate after 20 min. Figure 2b shows the effect of the partial replacement of gypsum by hemihydrate and Na_2_SO_4_ in the C_3_A-gypsum system on the precipitated ettringite. Substituting hemihydrate for 10% gypsum resulted in a significant decrease in the amount of precipitated ettringite, whereas substituting hemihydrate for 20% gypsum resulted in a slight change in the amount of ettringite in the first 20 min and a significant increase after 20 min. In addition, it is extremely remarkable that the rate of ettringite precipitation in first 20 min was greatly promoted by incorporating a small amount of Na_2_SO_4_ in the C_3_A-gypsum system.

### 3.2. Effect of Sulfates on SSA along C_3_A Hydration Reaction

**Figure 3:**
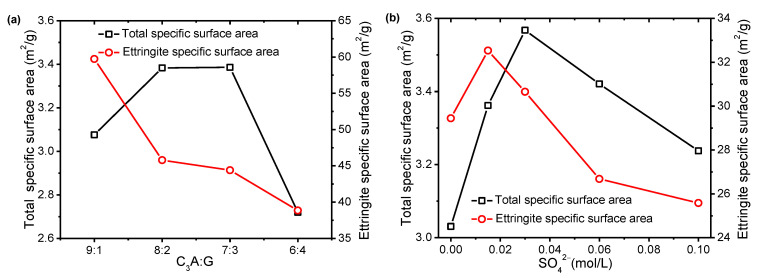
Specific surface area at the first 20 min: (**a**) effect of C_3_A/G; (**b**) effect of the SO_4_^2−^ concentration in simulated solutions.

Figure 3 shows the effect of C_3_A/G and the SO_4_^2−^ concentration in simulated solutions on the SSA at 20 min. Although the SSA of the initial powders gradually decreased with increasing gypsum, the SSA of the hydrated samples at 20 min increased first and then decreased. For the C_3_A hydration in simulated solutions with different SO_4_^2−^ concentrations, the SSA first increased and then decreased with the increasing SO_4_^2−^ concentration. The SSA reached the maximum in the simulated solution with a SO_4_^2−^ concentration of 0.03 mol/L, whereas the amount of ettringite reached the maximum at a SO_4_^2−^ concentration of 0.06 mol/L. The above-mentioned change trends of the SSA were not consistent with the formation process of ettringite. Considering that the change of SSA under the experimental conditions was mainly caused by the precipitation of ettringite, it was considered that the changes in the morphology of ettringite and the formation amount together resulted in the changes in SSA.

**Figure 4:**
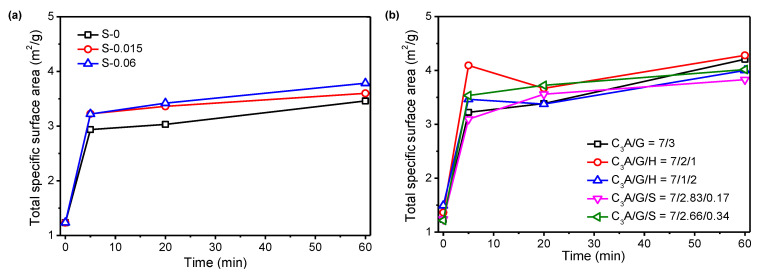
Specific surface area along C_3_A hydration: (**a**) effect of SO_4_^2−^ concentration in simulated solution; (**b**) effect of hemihydrate and Na_2_SO_4_ in solid.

Figure 4 shows the evolution of the SSA of hydrated samples along the C_3_A hydration. As expected, the SSA of hydrated samples increased significantly within the first 5 min. However, obvious differences were found after 5 min for different systems. For hydration in simulated solutions with different SO_4_^2−^ concentrations, the SSA of hydrated samples increased along the C_3_A hydration. As the SO_4_^2−^ concentration in simulated solution increased from 0 to 0.015 mol/L and 0.06 mol/L, the SSA of hydrated samples significantly increased. By replacing gypsum with quality hemihydrate of 10% and 20%, it could be clearly found that the SSA decreased at 20 min and then increased. Additionally, the higher hemihydrate that was added, the more significant this trend of change was. When a trace amount of sodium sulfate was added (1.7% and 3.4% of the total solid), the SSA gradually increased with the extension of hydration time. However, the above-mentioned change trends of the SSA were not consistent with the formation process of ettringite. This again indicated that the morphology of ettringite was variable. In addition, when hemihydrate was added, the change in SSA may also be related to the precipitated gypsum, since hemihydrate that reacted quickly with water could form gypsum [29]. This will be discussed in detail in the following section.

### 3.3. Effect of Sulfates on SSA and Morphology of Ettringite

According to the SSA of hydrated samples at *t* = 0, 5, 20 and 60 min and the amount of precipitated ettringite, the SSA of ettringite at *t* = 0, 5, 20 and 60 min could be calculated based on the following formula:

(1) $$S_{e}(t)=\frac{S(t)-(1-f(t))\times S(t_{0})}{f(t)}$$

where *f(t)* is the weight fraction of ettringite in the hydrated sample deduced from the DSC measurement, *S(t*_0_*)* is the SSA of the hydrated sample measured by BET at *t =* 0 and *S (t)* is the SSA of the hydrated sample at *t* measured by BET. It should be noted that there was an assumption for this calculation that the SSA of the anhydrous part in the hydrated sample was constant and equal to *S(t*_0_*)*.

The effect of C_3_A/G and the SO_4_^2−^ concentration in simulated solutions on the SSA of the ettringite at 20 min is show in Figure 3. It can be seen clearly that as the proportion of gypsum increased from 10% to 20%, 30% and 40%, the SSA of ettringite gradually decreased from 59.7 m^2^/g to 45.8 m^2^/g, 44.4 m^2^/g and 38.9 m^2^/g, respectively. With the increase in the SO_4_^2−^ concentration in simulated solutions, the SSA of ettringite first increased and then decreased. When the SO_4_^2−^ concentration is 0.015 mol/L, the specific surface area of ettringite formed in the simulated solution reached a maximum of 32.5 m^2^/g. Comparing the SSA of ettringite in the two systems, it was confirmed that the SSA of ettringite formed in the simulated solution was significantly lower than that formed in the deionized water.

**Figure 5:**
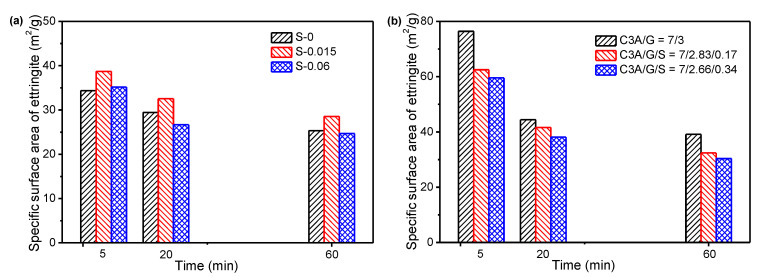
Specific surface area of ettringite precipitating along the C_3_A hydration: (**a**) effect of SO_4_^2−^ concentration in simulated solution; (**b**) effect of Na_2_SO_4_ in solid.

Figure 5 shows the evolution of the SSA of precipitated ettringite along the C_3_A hydration. It can be clearly seen that the SSA of ettringite gradually decreased along the C_3_A hydration reaction, which agreed well with results reported by Pourchet [13]. Taking C_3_A/G = 70/30 as an example, the SSA of ettringite at the very beginning of the C_3_A hydration (5 min) was about 76.4 m^2^/g, and decreased to 44.4 m^2^/g and 39.1 m^2^/g at 20 min and 60 min.

The concentration of SO_4_^2−^ in the simulated solution and the addition of Na_2_SO_4_ in the solid had a significant effect on the SSA of ettringite. For ettringite formed in simulated solutions with different SO_4_^2−^ concentrations, as the SO_4_^2−^ concentration in simulated solutions was 0, 0.015 mol/L and 0.06 mol/L, the SSA of ettringite at the very beginning of the C_3_A hydration (5 min) was 34.4 m^2^/g, 38.7 m^2^/g and 35.2 m^2^/g, respectively. For the C_3_A hydration at 20 min, the SSA of ettringite was 29.4 m^2^/g, 32.5 m^2^/g and 26.7 m^2^/g, respectively. For the C_3_A hydration at 60 min, the SSA of ettringite decreased to 25.3 m^2^/g, 28.5 m^2^/g and 24.7 m^2^/g, respectively. In other words, the SSA of ettringite in the simulated solution with a SO_4_^2−^ concentration of 0.015 mol/L was slightly higher than that in the simulated solution without SO_4_^2−^, but as the SO_4_^2−^ concentration increased to 0.06 mol/L, the SSA of ettringite was similar to that in the simulated solution without SO_4_^2−^. The SSA of ettringite could be significantly reduced by adding a small amount of Na_2_SO_4_ in a solid to replace gypsum. As the Na_2_SO_4_ content was 0, 1.7% and 3.4%, the SSA of ettringite at the very beginning of the C_3_A hydration (5 min) was 76.4 m^2^/g, 41.6 m^2^/g and 38.1 m^2^/g, respectively. For the C_3_A hydration at 20 min, the SSA ettringite was 44.4 m^2^/g, 41.6 m^2^/g and 38.1 m^2^/g, respectively. At 60 min of C_3_A hydration, the specific surface area of ettringite decreased to 39.1 m^2^/g, 32.4 m^2^/g and 30.34 m^2^/g, respectively. It was extremely obvious that the higher the Na_2_SO_4_ content was, the smaller the SSA of ettringite was. In addition, comparing the C_3_A hydration in different solution environments (deionized water and simulated solution), it could be seen that the SSA of ettringite in the simulated solution was significantly lower than that in deionized water. Taking C_3_A/G = 7/3 and S-0 as examples, the SSA of ettringite formed in deionized water at 5 min, 20 min and 60 min was 122%, 41% and 51% higher, respectively, than that of ettringite formed in the simulated solution. Considering that the precipitation reaction of gypsum might occur as hemihydrate was added, which resulted in a change in the SSA of the hydrated samples, it was difficult to accurately calculate the SSA of ettringite in this system.

**Figure 6:**
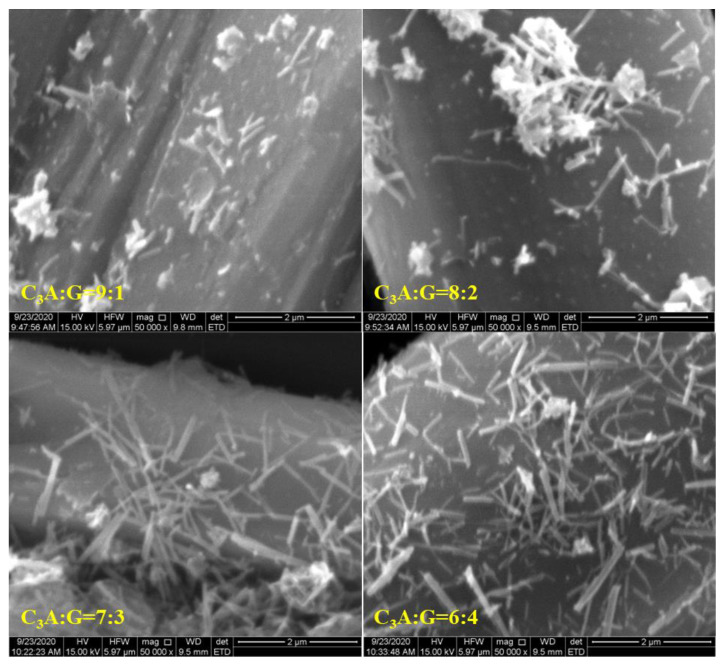
Effect of the proportion of gypsum in the C_3_A-gypsum system on morphology of precipitated ettringite.

**Figure 7:**
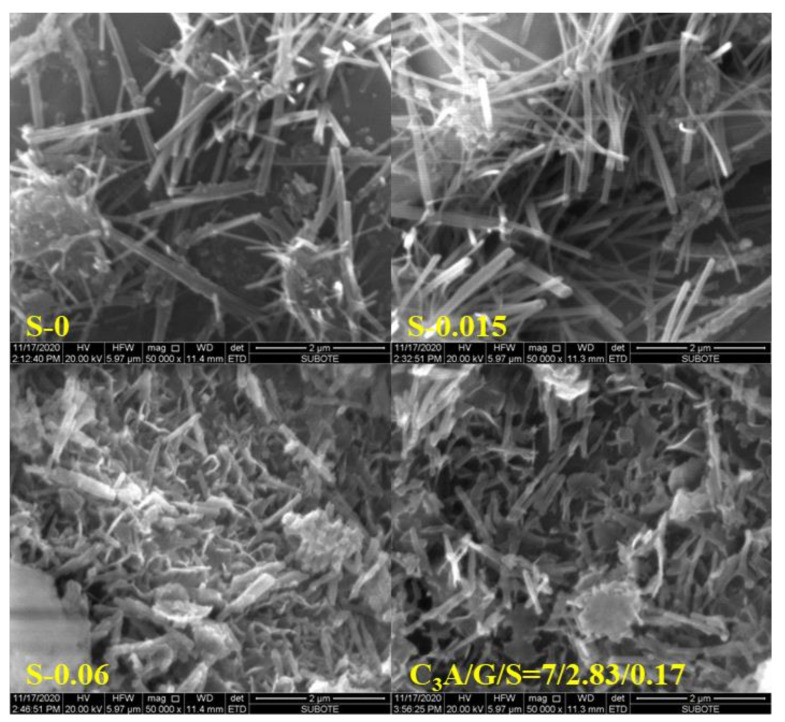
Effect of SO_4_^2−^ concentration in simulated solution and Na_2_SO_4_ in solid on morphology of precipitated ettringite.

**Figure 8:**
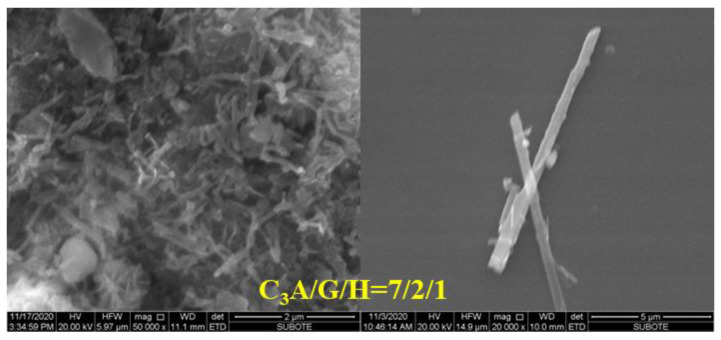
Morphology of precipitated ettringite and gypsum in the C_3_A-gypsum systems with hemihydrate.

The change of the SSA of ettringite must be caused by its morphology; therefore, the morphology of ettringite was observed via SEM and the results are shown in Figure 6, Figure 7 and Figure 8. As the proportion of gypsum in the C_3_A-gypsum systems increased, the diameter of the needle-shaped ettringite slightly increased. For the system with C_3_A/G of 70/30 in the simulated solution, ettringite was longer and larger in diameter when the SO_4_^2−^ concentration was 0 and 0.015 mol/L. However, when the SO_4_^2−^ concentration in the simulated solution was increased to 0.06 mol/L, the ettringite with a larger diameter but a shorter length was found. It can be seen clearly from Figure 8 that two processes of ettringite precipitation and gypsum precipitation occurred as hemihydrate was added during the hydration process of the C_3_A-gypsum system. It was consistent with the previous speculation. The long, rod-shaped gypsum particle was found, but its length and width were several times that of ettringite.

For all the samples, except those with hemihydrate, the size of ettringite at the very beginning was small; therefore it had a larger SSA, resulting in the great increase in the SSA of the hydrated sample within 5 min. Along the C_3_A hydration reaction, the formation of ettringite increased, and the total SSA of the hydrated sample continued to increase. However, the slowing down of the formation rate of ettringite and the growth of ettringite led to the decrease in its SSA, and both resulted in the slow growth of the SSA in the hydrated sample. For the samples with hemihydrate, the large increase in the SSA at the very beginning could be considered to be caused by the precipitation of ettringite and gypsum. However, with the increase in the amount of precipitated gypsum and rapid growth, the proportion of gypsum in the hydrated sample increased. Due to its larger size, the SSA of the hydrated sample decreased. However, the SSA of the hydrated sample would continue to increase with the increase in precipitated ettringite as the C_3_A hydration continued.

However, it should be noted that it was still extremely difficult to answer how sulfate affects the nucleation and growth of ettringite crystals. It had been suggested that the growth or nucleation of ettringite was closely related to the surface energy of ettringite. For a higher surface energy, ettringite tended to grow rather than nucleate; thus, the size was larger and its SSA was smaller.

Pourchet et al. [22] proposed that the PC in solution could make complex ions and/or reduce the surface energy of ettringite, which is beneficial to homogeneous nucleation, and that the PC strongly interacted with ettringite crystals and slowed down/prevented their growth. For this study, it was suggested that the surface energy of ettringite in the simulated solution was higher, and thus, the size was larger and the specific surface area was smaller. Adding Na_2_SO_4_ in a solid and increasing the proportion of gypsum could significantly increase the ionic strength of the solution. Therefore, ettringite had a higher surface energy and a larger specific surface area.

The amount and morphology of ettringite will affect the inter-particle interaction force [30], which eventually affects the rheological properties of cement paste. Therefore, further studies should be put forward on how the hydration of C_3_A-gypsum acts on the time-dependent rheological properties of cement paste.

## 4. Conclusions

Early hydration of the C_3_A-gypsum system was systematically studied in the presence of various types of sulfates such as gypsum, hemihydrate and Na_2_SO_4_ in the first hour. The BET method using nitrogen combined with a DSC analysis enabled us to quantitatively characterize the amount of precipitated ettringite and its specific surface area along the hydration. Based on the experimental results, the following conclusions can be drawn:

1. A large part of the ettringite precipitated within the first 20 min of the hydration reaction, whereas this reaction became very slow after 20 min. The amount of ettringite precipitated increased first and then decreased with the increasing proportion of gypsum in the C_3_A-gypsum system and SO_4_^2−^ concentration in the simulated solution. The rate of precipitation of ettringite in first 20 min was greatly promoted by incorporating a small amount of Na_2_SO_4_ in a solid in the C_3_A-gypsum system.
2. The specific surface area of the hydrated sample strongly increased within the first 5 min, but obvious differences were found when different sulfates were added after 5 min. The specific surface area of all the C_3_A-gypsum systems, except those with hemihydrate, slowly increased after 5 min, whereas the specific surface area of the C_3_A-gypsum system with hemihydrate decreased at 20 min and then increased.
3. The specific surface area of ettringite gradually decreased along the C_3_A hydration reaction. For all the C_3_A-gypsum systems, except those with hemihydrate, both the slowing down of the precipitation of ettringite and the growth of ettringite resulted in the slow growth of the specific surface area after 5 min. For the C_3_A-gypsum system with hemihydrate, two processes of ettringite precipitation and gypsum precipitation occurred. The nucleation and growth of ettringite and gypsum resulted in the complex changes in specific surface area, which first increased at the very beginning, then decreased and, finally, increased. In addition, different hydration environments had a very significant effect on the morphology of ettringite. The specific surface area of ettringite in the simulated solution was significantly lower than that in deionized water. The specific surface area of ettringite could be significantly reduced by adding a small amount of Na_2_SO_4_ in a solid.

## Figures and Tables

**Table 1:** The compositions of the simulated solutions.

Code	Na_2_SO_4_ (mol/L)	NaNO_3_ (mol/L)	Na^+^ (mol/L)	SO_4_^2−^ (mol/L)
S-0	0	0.2	0.2	0
S-0.015 mol/L	0.015	0.17	0.2	0.015
S-0.03 mol/L	0.03	0.14	0.2	0.03
S-0.06 mol/L	0.06	0.08	0.2	0.06
S-0.1 mol/L	0.1	0	0.2	0.1

**Table 2:** Mixture proportions and codes for C_3_A hydration at 20 min.

Code	C_3_A%	G%	PC/%	Mix Water
C_3_A/G = 90/10	90	10	0.1	Deionized water
C_3_A/G = 80/20	80	20	0.1	
C_3_A/G = 70/30	70	30	0.1	
C_3_A/G = 60/40	60	40	0.1	
S-0	70	30	0.1	S-0
S-0.015	70	30	0.1	S-0.015
S-0.03	70	30	0.1	S-0.03
S-0.06	70	30	0.1	S-0.06
S-0.1	70	30	0.1	S-0.1

**Table 3:** Mixture proportions and codes for C_3_A hydration in the first hour.

Code	C_3_A/%	G/%	H/%	S/%	PC/%	Mix Water
S0	70	30	0	0	0.1	S0
S0-0.015	70	30	0	0	0.1	S0-0.015
S0-0.06	70	30	0	0	0.1	S0-0.06
C_3_A/G = 70/30	70	30	0	0	0.1	Deionized water
C_3_A/G/H = 70/20/10	70	20	10	0	0.1	
C_3_A/G/H = 70/10/20	70	10	20	0	0.1	
C_3_A/G/S = 70/28.3/1.7	70	28.3	0	1.7	0.1	
C_3_A/G/S = 70/26.4/3.4	70	26.4	0	3.4	0.1	

## Data Availability

Not applicable.
